# Markers of Epithelial to Mesenchymal Transition in Association with Survival in Head and Neck Squamous Cell Carcinoma (HNSCC)

**DOI:** 10.1371/journal.pone.0094273

**Published:** 2014-04-10

**Authors:** Eirini Pectasides, Theodoros Rampias, Clarence Sasaki, Christos Perisanidis, Vassilis Kouloulias, Barbara Burtness, Thomas Zaramboukas, David Rimm, George Fountzilas, Amanda Psyrri

**Affiliations:** 1 Department of Internal Medicine (Medical Oncology), Yale University School of Medicine, New Haven, Connecticut, United States of America; 2 Department of Surgery (Otolaryngology), Yale University School of Medicine, New Haven, Connecticut, United States of America; 3 Department of Cranio-, Maxillofacial and Oral Surgery, Medical University of Vienna, Vienna, Austria; 4 Departments of Radiotherapy and Internal Medicine (Medical Oncology), Attikon University Hospital, National Kapodistrian University of Athens, Athens, Greece; 5 Department of Medical Oncology, Fox Chase Cancer Center, Philadelphia, Pennsylvania, United States of America; 6 Department of Pathology, Aristotle University of Thessaloniki School of Medicine, Thessaloniki, Greece; 7 Department of Pathology, Yale University School of Medicine, New Haven, Connecticut, United States of America; 8 Department of Medical Oncology, Aristotle University of Thessaloniki School of Medicine, Thessaloniki, Greece; Karolinska Institutet, Sweden

## Abstract

**Background:**

Elucidating the molecular phenotype of cancers with high metastatic potential will facilitate the development of novel therapeutic approaches to the disease. Gene expression profiles link epithelial to mesenchymal transition (EMT) phenotype with high-risk HNSCC. We sought to determine the role of protein biomarkers of EMT in head and neck squamous carcinoma (HNSC) prognosis.

**Methods:**

Protein expression analysis of EGFR, β-catenin and E-cadherin was performed on a cohort of 102 patients with HNSCC recruited between 1992 and 2005 using automated quantitative protein analysis (AQUA). We evaluated associations with clinicopathological parameters and prognosis.

**Results:**

There were 67 patients with primary squamous cell carcinoma of the head and neck in this cohort who met inclusion criteria and for whom we had complete E-cadherin, beta-catenin and EGFR expression data. High E-cadherin expressers had longer 5-year progression-free survival (PFS) compared to those with low E-cadherin (59.7% versus 40.6%, p = 0.04) and overall survival (OS) (69.6% versus 44.3%, p  = 0.05). Kaplan-Meier analysis showed that patients with low beta-catenin-expressing tumors trended toward worse 5-year PFS (p = 0.057). High EGFR expressers had inferior OS compared to low EGFR expressers (27.7% vs. 54%, p = 0.029). In the multivariable analysis context, E-cadherin remained an independent predictor of improved OS (HR = 0.204, 95% CI 0.043 to 0.972, p = 0.046) while EGFR trended towards significance for OS.

**Conclusions:**

The putative markers of EMT defined within a panel of HNSCC using AQUA are associated with tumors of poor prognosis.

## Introduction

Metastasis of head and neck squamous cell carcinoma (HNSCC) is a significant healthcare problem leading to high mortality rates. At present, TNM staging at presentation remains the most powerful tool for estimating the course of the disease. However, the accuracy of established methods of prognostic classification is often questionable and certainly insufficient for the design of personalized treatment strategies. Elucidating the molecular phenotype of cancers with high metastatic potential will facilitate the development of novel therapeutic approaches to the disease.

The evolving neoplastic clone accumulates genetic and epigenetic alterations that lead to genotypic and subsequently phenotypic changes that are subjected to selection for tumor progression. Among the numerous epigenetic alterations that have been characterized during malignant progression, epithelial to mesechymal transition is particularly interesting. For epithelial malignancies, the epithelial-mesenchymal transition (EMT) is considered to be the crucial event in the metastatic process. Epithelial-mesenchymal transition (EMT) is defined as loss of epithelial morphology and acquisition of migratory mesenchymal features such as changes in the morphology to spindle-shaped and motile fibroblastoid phenotype and loss of tight- and adherens-junction proteins, which allows the tumor cells to pass through the basement membrane and travel to the site of metastasis formation without being affected by conventional treatment. EMT has been associated with advanced stage of tumor progression and metastasis. The genetic instability often triggers alterations in gene expression regulation and accelerates the acquisition of EMT phenotype in carcinomas. Gene expression profiles link epithelial to mesenchymal transition (EMT) phenotype with high-risk HNSCC. EMT has become clinically significant due to the association with resistance to epidermal growth factor receptor tyrosine kinase inhibitors, such as erlotinib in lung cancer. The better understanding of EMT is important as targeting of this process with novel drugs can retard malignant progression or reverse resistance to EGFR-targeted therapies.

EMT is characterized by the combined loss of epithelial cell junction proteins such as E-cadherin and the acquisition of mesenchymal markers such as vimentin. Loss of E-cadherin protein expression is regarded as a hallmark of EMT, and leads to disassembly of adherens junctions, increased tumor cell motility and invasiveness. Blockade of E-cadherin in vitro, leads to the acquisition of spindle morphology and de novo expression of vimentin, features consistent with epithelial-to-mesenchymal transition[1].

Deregulated signaling through signal transduction pathways such phosphatidylinositol 3'-kinase (PI3K) pathway or Wnt pathway appear to mediate EMT. Pi3Ka activates the Akt1 and Akt2 Ser/Thr kinase, responsible for proliferation and antiapoptotic function. In the present study, we correlated the protein expression of EMT markers such as E-cadherin, beta-catenin and EGFR on a HNSCC tissue microarray annotated with patient follow-up data with survival outcomes using automated quantitative protein analysis.

## Patients and Methods

### Ethics Statement

The research was approved by the Yale University and the Aristotle University Hospital Institutional Review Boards, and written informed consent was obtained from each patient involved in the study.

### Patient population

Inclusion criteria were histologically confirmed primary squamous cell carcinomas of the head and neck treated at Yale-New Haven Hospital and Aristotle University Hospital of Thessaloniki between 1992 and 2005, and therapy with either external beam radiotherapy (EBRT) or gross total surgical resection and postoperative radiotherapy. Exclusion criteria included presentation with metastatic or recurrent disease or failure to receive a full course of radiation therapy.

### Tissue microarray construction

A tissue microarray consisting of tumors from each patient in the cohort was constructed at the Yale University Tissue Microarray Facility. Following institutional review board approval, the tissue microarray was constructed as previously described[2], including 102 cases. Tissue cores 0.6 mm in size were obtained from paraffin-embedded formalin-fixed tissue blocks from the archives of the Yale University and Aristotle University of Thessaloniki Department of Pathology. Hematoxylin- and eosin-stained slides from all blocks were first reviewed by a pathologist to select representative areas of invasive tumor to be cored. The cores were placed on the recipient microarray block using a Tissue Microarrayer (Beecher Instrument, Silver Spring, MD). All tumors were represented with two-fold redundancy. Previous studies have demonstrated that the use of tissue microarrays containing one to two histospots provides a sufficiently representative sample for analysis by immunohistochemistry. Addition of a duplicate histospot, while not necessary, does provide marginally improved reliability [3]. Cores from HPV16-positive SiHa cell lines fixed in formalin and embedded in paraffin were selected for positive controls and included in the array. Additionally, 10 histologically confirmed normal squamous epithelium samples from formalin-fixed and paraffin-embedded skin were included for comparison of E-cadherin, beta-catenin and EGFR expression in normal tissue. The tissue microarray was then cut to yield 5-µm sections and placed on glass slides using an adhesive tape transfer system (Instrumedics Inc., Hackensack, NJ) with UV cross-linking.

### Quantitative Immunohistochemistry

Tissue microarrays were deparaffinized and stained as previously described [4]. In brief, slides were deparaffinized with xylene and rehydrated through changes of ethanol with decreasing concentrations. Slides were then subjected to heat induced antigen retrieval by pressure cooking in 0.1 mol/L citrate buffer (pH 6.0) for approximately 10 min. Endogenous peroxidase activity was blocked by incubating in 0.3% hydrogen peroxide in methanol for 30 min. Nonspecific antibody binding was blocked with 0.3% bovine serum albumin for 30 min at room temperature. Following these steps, slides were incubated with mouse monoclonal primary antibody to E-cadherin (1∶1000, #610181, BD Transduction Laboratories, San Jose, CA), beta-catenin (1∶4000, #610153, BD Transduction Laboratories, San Jose, CA), EGFR (1∶500, clone 31G7, Zymed Laboratories, San Francisco, CA) or p16 (1∶25, #9517, MTM Labotories, Westborough, MA) at 4°C overnight. These antibodies have been extensively validated in previous studies using immunohistochemistry and Western blot analysis of neoplastic tissue and tumor cell lines [5]–[7]. Subsequently, slides were incubated with goat anti-mouse secondary antibody conjugated to a horseradish peroxidase-decorated dextran polymer backbone (Envision, Dako Corporation, Carpinteria, CA) for 1 hr at room temperature. Tumor cells were identified by use of anticytokeratin antibody (rabbit anti-pancytokeratin antibody, 1∶100, Z0622, Dako Corporation, Carpinteria, CA) with subsequent goat anti-rabbit antibody conjugated to Alexa 546 fluorophore (1∶100, A11035, Molecular Probes, Eugene, OR). We added 4',6-diamidino-2-phenylindole (DAPI) to visualize nuclei (Prolong Gold with DAPI, P36931, Molecular Probes, Eugene, OR). Fluorescent chromogen Cy-5 tyramide (1∶50, Perkin Elmer Corp, Wellesley, MA) was used for target identification. Cy-5 (red) was used because it is well outside the green-orange spectrum of tissue autofluorescence.

### Automated Image Acquisition and Analysis

Automated image acquisition and analysis using automated in situ quantitative measurement of protein analysis (AQUA) has been described previously [6]. In brief, monochromatic, high-resolution (1,024×1,024 pixel; 0.5 µm) images were obtained of each histospot. We distinguished areas of tumor from stromal elements by creating a mask from the cytokeratin signal. 4',6-Diamidino-2-phenylindole signal was used to identify nuclei, and the cytokeratin signal was used to define cytoplasm. Overlapping pixels (to a 99% confidence interval) were excluded from both compartments. The signal (AQUA score) was scored on a normalized scale of 0 to 255 expressed as pixel intensity divided by the target area. AQUA scores for each subcellular compartment (nuclear and cytoplasmic) as well as the tumor mask were recorded. AQUA scores for duplicate tissue cores were averaged to obtain a mean score for each tumor.

### Statistical Analysis

Histospots containing <5% tumor as assessed by mask area (automated) were excluded from further analysis. Pearson’s correlation coefficient (R) was used to assess the correlation between log AQUA scores from redundant tumor cores and Spearman rank correlation (*ρ*) to assess the association between E-cadherin, beta-catenin and EGFR. Survival analysis was performed at 5-year cutoffs. Progression-free survival and overall survival were assessed by Kaplan-Meier analysis with log-rank score for determining statistical significance. Relative risk was assessed by Cox proportional hazards regression by multivariable analysis. Correlations between clinicopathologic characteristics and survival were assessed by univariate Cox regression. All calculations and statistical analyses were performed by SPSS 15.0 for Windows (SPSS, Inc., Chicago, IL).

## Results

### Clinical and Pathologic Variable Analysis

There were 67 patients with primary squamous cell carcinoma of the head and neck in this cohort who met inclusion criteria and for whom we had complete E-cadherin, beta-catenin and EGFR expression data. We excluded from the analysis 35 cases that had missing expression information. Fifty-six (84%) of the patients were male and 11 (16%) were female. Five (7%) patients were tumor-node-metastasis (TNM) stage I, 9 (13%) stage II, 17 (25%) stage III, 32 (48%) stage IV, and 4 were not recorded. Tumor sites included 7 (10%) oral cavity, 28 (42%) larynx, 24 (36%) pharynx, 2 (3%) hypopharynx, and 6 were not recorded. For histologic grade, 9 (13%) tumors were well differentiated, 28 (42%) were moderately differentiated, 20 (30%) were poorly differentiated, and 10 were not recorded. Seven of 49 tumors available for HPV analysis by in situ hybridization were HPV+, all of which were oropharyngeal tumors. No significant correlation was found between the clinical and pathologic characteristics and outcome, as assessed by univariate Cox regression. These data are summarized in Table 1.

**Table 1:** Demographic, clinical and pathologic characteristics.

Variable	No	%	Hazard Ratio	95% CI	*P*
Gender					
Male	56	84			
Female	11	16	0.71	0.21 – 2.37	0.57
TNM stage					
I	5	7			
II	9	13	0.76	0.11 – 5.41	0.79
III	17	25	0.70	0.13 – 3.87	0.69
IV	32	48	1.57	0.35 – 6.92	0.55
Unknown	4				
Tumor Site					
Oral cavity	7	10			
Larynx	28	42	1.37	0.36 – 5.26	0.65
Oropharynx	24	36	0.97	0.26 – 3.67	0.97
Hypopharynx	2	3	0.92	0.10 – 8.86	0.94
Unknown	6				
Tumor grade					
Well differentiated	9	13			
Moderately differentiated	28	42	0.58	0.15 – 2.15	0.41
Poorly differentiated	20	30	0.47	0.11 – 2.00	0.3
Unknown	10				
HPV status					
Negative	42	63			
Positive	7	10	0.54	0.13 – 2.29	0.4
Unknown	18	27			

### Quantitative Immunohistochemistry for E-cadherin Protein Expression

**Figure 1:**
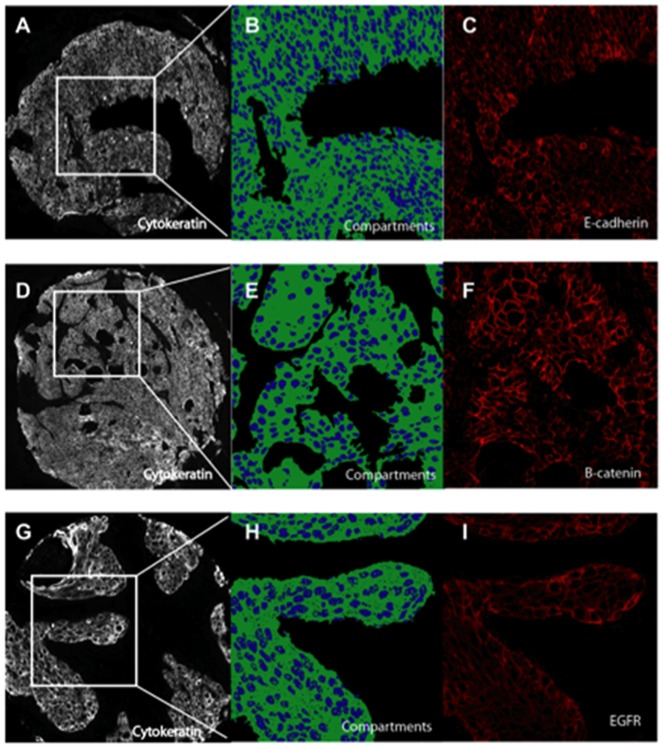
Fluorescent immunohistochemistry for automated analysis (AQUA). A, D, G. Cytokeratin was used to identify tumor within each histospot. B, E, H. Pseudocolored colocalization image demonstrating compartment assignment. Cytokeratin-Cy3 (green) was used to define the non-nuclear compartment; DAPI (blue) was used to define the nuclear compartment. C, F, I. Cy5 (red) was used to identify E-cadherin (C), beta-catenin (F) and EGFR (I).

As visualized by fluorescent immunohistochemistry, E-cadherin displayed membranous and cytoplasmic staining (Figure 1; A–C). Tumor E-cadherin expression followed a skewed distribution as expected for a cancer tissue biomarker (Figure S1a). AQUA scores under the tumor mask were averaged between the two histospots and final scores ranging from 6.4 to 74.82 were obtained for 67 patients. To assess for intratumor heterogeneity of E-cadherin expression and control for reproducibility of the assay, we compared AQUA scores from redundant tumor cores and observed significant correlation (Figure S2a; R = 0.89). The cohort was divided in high (>75^th^ percentile) and low E-cadherin expressers (<75^th^ percentile), based on a previously published study [5].

### Quantitative Immunohistochemistry for beta-catenin Protein Expression

beta-catenin showed a mixed membranous and cytoplasmic expression pattern (Figure 1; D-F), following a skewed distribution (Figure S1b). AQUA scores ranged from 3.81 to 83.45 and intratumor heterogeneity for beta-catenin was low, as assessed by Pearson’s R (Figure S2b; R = 0.85). The cohort was again split in high and low beta-catenin expressers using the 75^th^ percentile as the cutpoint [6].

### Quantitative Immunohistochemistry for EGFR Protein Expression

The staining pattern for EGFR was mainly membranous and cytoplasmic (Figure 1, G–I) and tumor AQUA scores ranged between 2.64 and 62.9 (Figure S1c). The regression between scores from double redundant spots was again high (Figure S2c; R = 0.89), which allowed us to average the two scores for each tumor and use the mean score in our analysis. The cohort was divided in high and low EGFR expressers using the median score as the cutpoint [8].

### Correlation between biomarkers and clinicopathologic variables

E-cadherin expression was higher in female patients (p = 0.038) and oropharyngeal tumors (p = 0.001). Oropharyngeal tumors were also associated with higher β-catenin (p<0.001) and p16 levels (p = 0.012). There was no other association between biomarkers and clinicopathologic features, including stage and histologic grade.

### Univariate Survival Analysis

#### Progression-free survival

**Figure 2:**
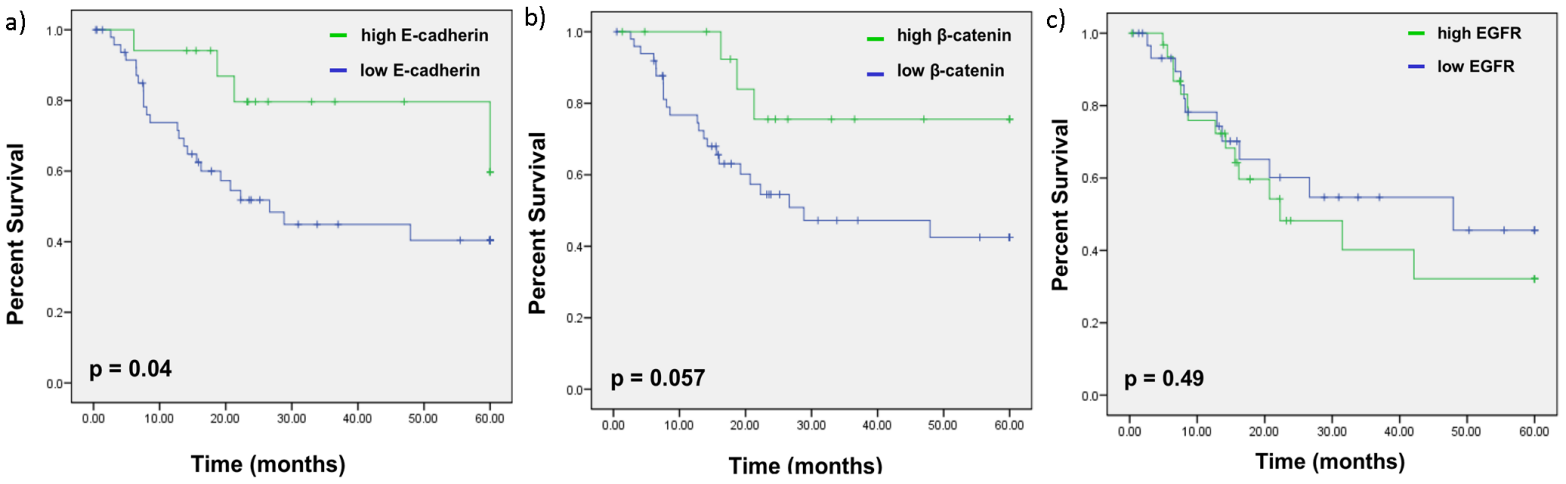
Kaplan-Meier survival curve comparing progression-free survival estimation between low- and high-expressing E-cadherin, beta-catenin and EGFR groups. a) Patients with high tumor E-cadherin expression exhibit a higher probability of PFS (59.7% vs. 40.6%, p = 0.04). b) Patients with low expression of beta-catenin trended towards worse PFS (p = 0.057). c) There was no association between EGFR expression and PFS (p = 0.49).

The expression status of E-cadherin, beta-catenin and EGFR were evaluated for association with progression-free survival (PFS) using Kaplan-Meier survival analysis with log-rank statistic to determine significance. This analysis demonstrates that high E-cadherin expression is associated with superior 5-year PFS. Patients with high E-cadherin had a PFS of 59.7% compared with 40.6% for patients with low E-cadherin (p = 0.04) (Figure 2a). Kaplan-Meier analysis showed that patients with low beta-catenin-expressing tumors trended toward worse 5-year PFS but that was not statistically significant (p = 0.057) (Figure 2b). However, there was no association between EGFR expression and PFS (p = 0.49) (Figure 2c).

#### Overall Survival

**Figure 3:**
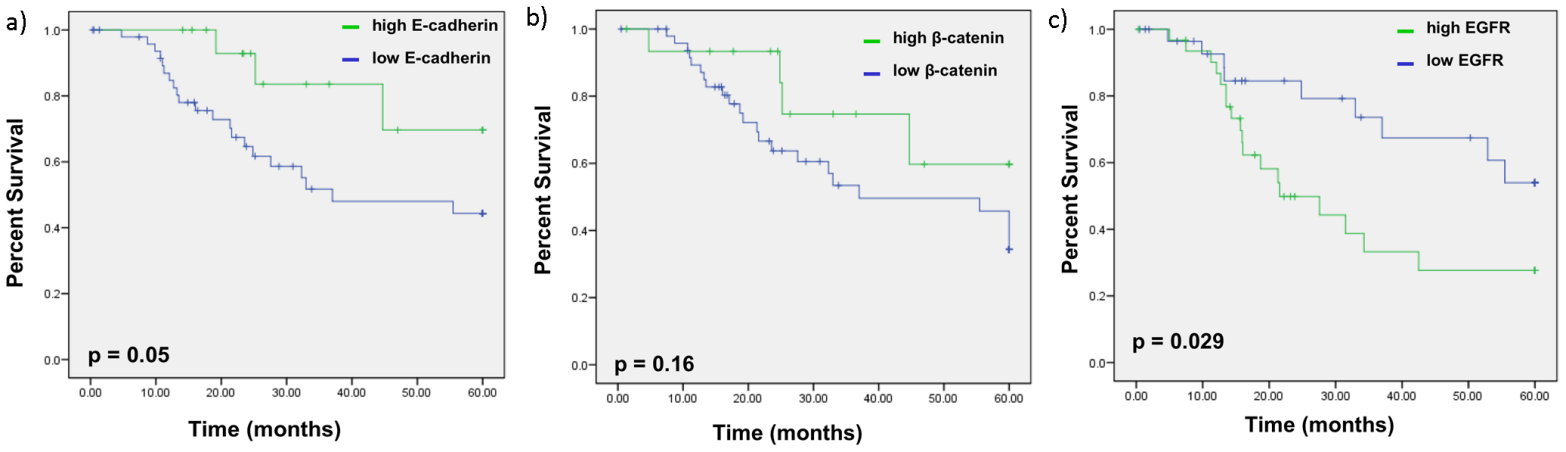
Kaplan-Meier survival curve comparing overall survival estimation between low- and high-expressing E-cadherin groups. a) Patients with high tumor E-cadherin expression exhibit a higher probability of OS (69.6% vs. 44.3%, p = 0.05). b) There was no association between beta-catenin and OS (p = 0.16). c) Patients with high tumor EGFR expression had inferior 5-year overall survival compared with those with low tumor EGFR expression (27.7% vs. 54%, p = 0.029).

The expression status of E-cadherin, beta-catenin and EGFR were also evaluated for association with OS. Kaplan-Meier analysis demonstrated that there was a significant correlation between high E-cadherin expression and improved OS. Patients with high E-cadherin expression had an OS of 69.6% compared with 44.3% for patients with low E-cadherin (p = 0.05) (Figure 3a). Kaplan-Meier analysis also revealed lack of any association for beta-catenin expression (p = 0.16) (Figure 3b). EGFR, however, was a significant prognostic factor, as high EGFR expressers had inferior OS compared with low EGFR expressers (27.7% vs. 54%, p = 0.029) (Figure 3c).

### Multivariable Survival Analysis

Using the Cox proportional hazards model, we carried out multivariable analysis to assess the prognostic value of E-cadherin and EGFR expression by AQUA for OS. We also included the following prognostic variables in the regression model: sex, TNM stage, tumor grade and tumor site. E-cadherin remained an independent predictor of improved OS (HR = 0.204, 95% CI 0.043 to 0.972, p = 0.046). High EGFR expression trended toward significance for OS (p = 0.07). Results of the multivariable analysis are summarized in Table 2. Adjusting for HPV status, neither E-cadherin nor EGFR predicted for OS (E-cadherin, HR = 0.257, 95% CI 0.034 to 1.937, p = 0.187; EGFR, HR = 0.382, 95% CI 0.042 to 3.445, p = 0.391).

**Table 2:** Multivariable 5-year overall survival (OS) analysis, by Cox regression.

Variable	Hazard Ratio	95% CI	*P*
Male gender			
Female gender	1.079	0.261–4.462	0.92
TNM Stage I			
TNM Stage II	1.485	0.158–14.008	0.73
TNM Stage III	1.532	0.201–11.689	0.681
TNM Stage IV	4.707	0.693–31.959	0.113
Histology, well differentiated			
Histology, moderately differentiated	0.601	0.136–2.663	0.503
Histology, poorly differentiated	0.819	0.155–4.337	0.814
Tumor Site, oral cavity			
Tumor Site, larynx	1.848	0.4–8.533	0.432
Tumor Site, oropharynx	2.146	0.511–9.006	0.297
EGFR AQUA Score (high versus low)	4.98	0.87–28.5	0.07
E-cadherin AQUA Score (high versus low)	0.204	0.043–0.972	0.046

## Discussion

The high mortality rate of locally advanced HNSCC has prompted not only the discovery of molecular alterations required to initiate invasion and metastasis, but also the evaluation of the occurrence of expression of these molecules in vivo as independent prognostic markers orthogonal to the commonly assayed clinical variables. EMT is linked with the loss of cell-cell adhesion, cellular elongation, and invasion of the underlying extracellular matrix. EMT progression is characterized by the loss of proteins involved in cell junctions such as E-cadherin and the claudins, and the expression of mesenchymal markers such as vimentin. The EMT appears to be controlled by signal-transduction pathways such as the Wnt signaling pathway which can be aberrantly activated during neoplasia. Also involved is PI3Kα, which activates the Akt1 and Akt2 Ser/Thr kinase, responsible for cell proliferation and inhibition of apoptosis.

In the present study, we have assessed the expression and prognostic value of a panel of three markers (EGFR, E-cadherin, beta-catenin) associated with the EMT phenotype.

We found that high E-cadherin protein level is an independent predictor of improved OS in HNSCC. A significant correlation between the expression of E-cadherin and beta-catenin was also observed. E-cadherin is a cell surface glycoprotein that mediates intercellular adhesion through hemophilic interactions of its extracellular domain and interactions of its cytoplasmic domain with beta-catenin and plakoglobin. Mutations and deletions of the cytoplasmic tail of E-cadherin which contains the beta-catenin binding site result in dissociation of cell-cell- adhesion complexes [9]. In addition, loss or downregulation of E-cadherin expression results in destabilization of cadherin/catenin complex formation leading to the disassembly of cellular adherens junctions. Therefore, loss or downregulation of E-cadherin expression is involved in epithelial-mesenchymal transition (EMT). Increased cell motility and invasiveness in E-cadherin negative tumors and cell lines have been attributed to the loss of cell-cell adhesion. Restoration of E-cadherin expression in cancer cells results in decreased invasiveness, growth suppression and terminal differentiation [10]–[12]. Loss or downregulation of E-cadherin results in release of beta-catenin from a membrane-bound state into cytoplasm. Cytoplasmic beta-catenin can translocate into the nucleus where is known to interact with transcription factors of the leukocyte enchancer factor (LEF)/T-cell factor (TCF) family to regulate transcription of target genes implicated in cell growth control such as cyclin D1 and c-myc [13], [14]. By sequestering beta-catenin at the cell surface, E-cadherin antagonizes beta-catenin signaling pathways and induces growth inhibition [15]. Previous studies have shown that E-cadherin expression can deplete the cytoplasmic beta-catenin levels inhibiting the nuclear beta-catenin signaling and cell growth in an adhesion-independent manner [13], [16]–[18].

We also found a positive correlation between E-cadherin and EGFR expression. It is well known that cadherins interact with growth factor receptors at the cell surface [19]–[23]. Numerous studies have reported that E-cadherin interact with EGFR through the core armadillo repeat domain of beta-catenin [24], [25]. Pece and Gutkind have described that cadherin clustering can recruit EGFR [21]. They also observed that the interaction of E-cadherin and EGFR resulted in increased MAPK activity. Similar observations regarding the regulation of MAPK activity by the EGFR/E-cadherin/catenin complexes were made by Qian and colleagues [24]. The epidermal growth factor receptor (EGFR) is overexpressed in head and neck squamous cell carcinoma [26]. It is possible, that the efficient formation of membrane EGFR/E-cadherin/catenin complexes in a high density is crucial for activation of MAPK signaling and cancer progression in head and neck epithelial cells. While EGFR overexpression is thought to be an early event in cancer development, the downregulation of E-cadherin may occur at later stages of cancer progression, thereby leading to tumor invasion and metastasis.

In the work by Boyer et al EMT was correlated with advanced stage of tumor progression and metastasis [27]. Chung et al [28], using cDNA microarray technology in 60 fresh frozen HNSCC samples categorized these tumors into distinct subtypes with statistically significant differences in recurrence-free survival. The most significant sets of genes enriched in the high-risk tumors were genes involving EMT, NF- B activation, and cell adhesion. This signature was validated in an independent cohort of 40 HNSCC and the RNA used was extracted from FFPE tumors. In addition, EMT has gained significant attention clinically in HNSCC and non-small cell lung carcinoma due to the association with resistance to epidermal growth factor receptor tyrosine kinase inhibitors, such as erlotinib [29]. In the past decade, the development of novel therapeutic strategies targeting specific tumor-associated proteins has included monoclonal antibodies directed against growth factor receptors and small molecules that inhibit tyrosine kinases. The epithelial growth factor receptor (EGFR) overexpressed in many cancers including HNSCC, gliomas, and lung, ovarian, and pancreatic cancers is one of these targeted molecules. Only a minority of patients with HNSCC respond to EGFR-directed inhibition, underscoring the need for predictive markers to select patients most likely to respond to small molecule EGFR-tyrosine kinase inhibitors (TKI) or monoclonal antibodies against EGFR. In addition, with the well-known involvement of Src kinase in EMT and the development of Src kinase inhibitors, such as AZ0530 and dasatinib, in clinical trials, the understanding of EMT has increased clinical significance and importance of EMT as a process that can be targeted by novel drugs. Mandal et al [30], studied eleven HNSC cell lines and 50 primary tumors using western blot analysis as well as immunohistochemical, and functional techniques to assess the status of activated Src (p-Src), E-cadherin, and vimentin in both cell lines and tumor tissues and the results were correlated with patients’ clinicopathologic parameters. The authors found an inverse expression of p-Src and E-cadherin in the majority of cell lines and primary tumors compared with normal squamous mucosa. Elevated levels of p-Src were associated with down-regulation of E-cadherin and the expression of vimentin in epithelial tumor cells. In vitro inhibition of Src resulted in E-cadherin re-expression and increased cell to cell contact in squamous carcinoma cell lines. Immunohistochemical analysis of these markers in primary tumors demonstrated a significant correlation between the EMT molecular phenotype (increased p-Src, decreased E-cadherin, and vimentin expression) and aggressive tumor features including penetrating invasive fronts, high-grade sarcomatoid transformation, and lymph node metastasis. Keysar et al [31] recently showed that EGFR-dependent HNSCC cells develop both EGFR-dependent and –independent EMT and Hedgehog signaling regulates both processes. The combination of Hedgehog inhibitor (IPI-926) with cetuximab could restore sensitivity to cetuximab and delay tumor recurrence.

Our data also confirm that HPV status is the most powerful prognostic factor in HNSCC. Our findings, therefore, should be validated in HPV negative cohorts.

To summarize, quantitative assessment of EMT-associated proteins on a TMA is feasible and can provide important prognostic information.

## Supporting Information

Figure S1 **Histograms of a) E-cadherin, b) beta-catenin and c) EGFR expression under the tumor mask.** AQUA analysis showed a left-skewed distribution, as expected for tissue biomarkers.
(TIFF)

Figure S2 **Linear regression of log-normalized AQUA scores for a) E-cadherin, b) beta-catenin and c) EGFR shows that agreement between two independent samples from each patient was high (E-cadherin,** ***R*** **= 0.79; beta-catenin,** ***R*** **= 0.85; EGFR,** ***R*** **= 0.89).**
(TIFF)
