# Supplementary figures and images for: Markers of Epithelial to Mesenchymal Transition in Association with Survival in Head and Neck Squamous Cell Carcinoma (HNSCC)
Source: PLoS One. 2014 Apr 10;9(4):e94273. doi: 10.1371/journal.pone.0094273 (PMC3983114; doi:10.1371/journal.pone.0094273)

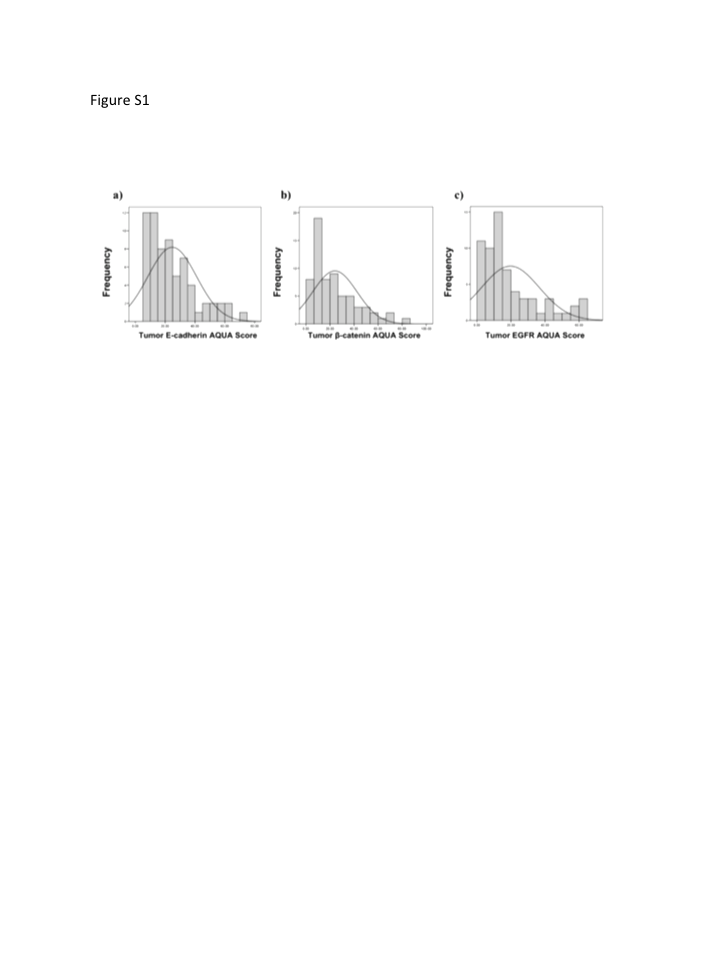

Supplement: Figure S1 — Histograms of a) E-cadherin, b) beta-catenin and c) EGFR expression under the tumor mask. AQUA analysis showed a left-skewed distribution, as expected for tissue biomarkers. (TIFF) [file pone.0094273.s001.tiff]

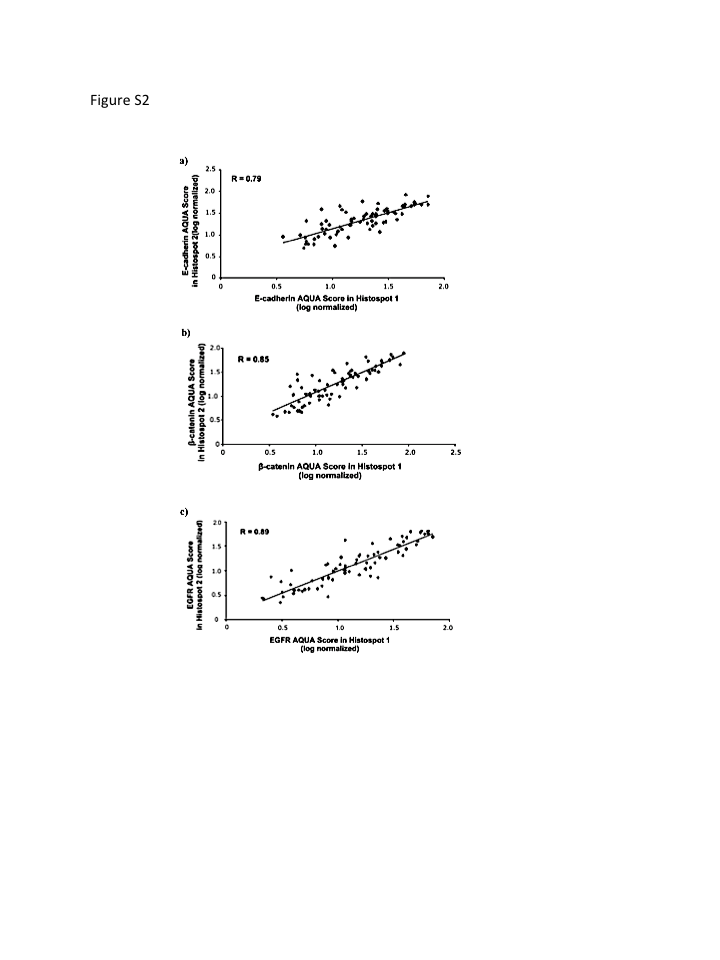

Supplement: Figure S2 — Linear regression of log-normalized AQUA scores for a) E-cadherin, b) beta-catenin and c) EGFR shows that agreement between two independent samples from each patient was high (E-cadherin, R = 0.79; beta-catenin, R = 0.85; EGFR, R = 0.89). (TIFF) [file pone.0094273.s002.tiff]
